# Impact of a spatial repellent intervention on *Anopheles kdr* insecticide resistance allele in Sumba, Indonesia

**DOI:** 10.1186/s12936-024-04841-1

**Published:** 2024-01-22

**Authors:** Lepa Syahrani, Puji B. S. Asih, Anom Bowolaksono, Astari Dwiranti, Siti Zubaidah, Ismail E. Rozi, Dendi H. Permana, Claus Bøgh, Michael J. Bangs, John P. Grieco, Nicole L. Achee, Neil F. Lobo, Din Syafruddin

**Affiliations:** 1 Doctoral Program, Department of Biology, Faculty of Mathematics and Natural Sciences, University of Indonesia, Depok, Indonesia; 2 Eijkman Research Center for Molecular Biology, National Research and Innovation Agency (BRIN), Cibinong, Indonesia; 3 Department of Biology, Faculty of Mathematics and Natural Sciences, University of Indonesia, Depok, Indonesia; 4 Doctoral Program, Faculty of Medicine, University of Hasanuddin, Makassar, Indonesia; 5 Doctoral Program of Biomedical Sciences, Faculty of Medicine, University of Indonesia, Jakarta, Indonesia; 6 The Sumba Foundation, Public Health and Malaria Control, Sumba, Indonesia; 7 Public Health and Malaria Control, PT Freeport Indonesia, International SOS, Mimika, Indonesia; 8 Department of Biological Sciences, Eck Institute for Global Health, University of Notre Dame, Indiana, USA; 9 Department of Parasitology, Faculty of Medicine, Hasanuddin University, Makassar, Indonesia; 10 Hasanuddin University Medical Research Center (HUMRC), Makassar, Indonesia

**Keywords:** *Kdr*, Pyrethroid, VGSC, L1014F, L1014S, Resistance

## Abstract

**Background:**

The emergence of insecticide resistance and outdoor transmission in malaria-endemic areas underlines the urgent need to develop innovative tools, such as spatial repellents (SR), that may circumvent this residual transmission. With limited options for effective insecticides, regular resistance monitoring is warranted for selecting and using appropriate tools. This study evaluates the pyrethroid knockdown resistance (*kdr*) allele before and after implementing a transfluthrin-based spatial repellent (SR) intervention in placebo-treated clusters.

**Methods:**

This study looks at the frequency distribution of the *kdr* allele in Sumba Island from June 2015 to August 2018. Insecticide susceptibility tests were carried out on female *Anopheles* sp. aged 3–5 days against permethrin 21.5 μg/ml, deltamethrin 12.5 μg/ml, and transfluthrin 10 μg/ml using CDC bottle assay. PCR sequencing of representative samples from adult mosquito collections and insecticide tests revealed the presence of *kdr* mutations (L1014F and L1014S) in the *VGSC* gene.

**Results:**

A total of 12 *Anopheles* species, *Anopheles tesselatus, Anopheles. aconitus, Anopheles barbirostris, Anopheles kochi, Anopheles annularis, Anopheles maculatus, Anopheles sundaicus, Anopheles flavirostris, Anopheles balabacensis, Anopheles indefinitus, Anopheles subpictus,* and *Anopheles vagus* were analysed. *Anopheles vagus* and *An. sundaicus* predominated in the larval populations. Susceptibility assays for all insecticides identified fully susceptible phenotypes in all species examined. *Anopheles* increasing frequency of *kdr* mutant alleles during the 3 year SR deployment was observed in both SR-treated and placebo areas, a statistically significant increase occurred in each arm. However, it is unclear how significant SR is in causing the increase in mutant alleles. The L1014S, knockdown resistance east type (*kdr-e)* allele was detected for the first time among the mosquito samples in this study. The L1014F, knockdown resistance west type (*kdr-w*) allele and heteroduplex form (wild-type—mutant) were found in almost all *Anopheles* species examined, including *An. vagus, An. aconitus, An. subpictus, An. tesselatus, An. annularis, An. flavirostris* and *An. sundaicus.*

**Conclusion:**

The presence of fully susceptible phenotypes over time, along with an increase in the frequency distribution of the L1014F/S mutations post-intervention, suggest drivers of resistance external to the study, including pyrethroid use in agriculture and long-lasting insecticidal nets (LLINs). However, this does not negate possible SR impacts that support resistance. More studies that enable the comprehension of possible SR-based drivers of resistance in mosquitoes need to be conducted.

**Supplementary Information:**

The online version contains supplementary material available at 10.1186/s12936-024-04841-1.

## Background

Malaria is still endemic in nine of the 11 WHO Southeast Asia Region countries, including Indonesia. The number of malaria cases, as indicated by the Annual Parasite Incidence (API), decreased to less than one from 2015 to 2020 but increased to 1.1 in 2021, with a positive trend of malaria cases seen in Eastern Indonesia, including Papua Province, West Papua Province, and East Nusa Tenggara Province [1]. The malaria control and elimination programme now relies mainly on two vector intervention tools, long-lasting insecticidal nets (LLINs) and indoor residual spraying (IRS), that use insecticides to mitigate malaria transmission. The emergence of *Anopheles* strains resistant to currently used insecticides poses a critical challenge to achieving malaria elimination by 2030 [2, 3]. Vector control is an essential aspect of the program to combat malaria transmitted by *Anopheles*. In Indonesia, insecticide use has been the mainstay in controlling many vector-borne diseases and other agricultural pests [4]. The effectiveness of mosquito control programmes relies heavily on the efficacy of insecticides used either on LLINs or IRS, bionomics of the target vector, and human behaviour [5, 6]. Spatial repellents (SR), which repel mosquitoes from humans, thereby preventing infectious bites, also diminish the emergence and spread of resistance since effective concentrations are sublethal and may not select for resistance alleles [7]. Historically, the SR paradigm has been widely used in Indonesian communities, with active ingredients (AIs) limited to pyrethroid compounds, such as metofluthrin and transfluthrin [8–10].

Currently used insecticides for vector control include pyrethroids (alpha-cypermethrin, bifenthrin, cyfluthrin, deltamethrin, and lambda-cyhalothrin), carbamates (bendiocarb, propoxur), and organophosphates (fenitrothion, malathion, pirimiphos–methyl) [4]. The use of pyrethroid is not only limited to vector control in public health but is also used to control agricultural pests. The widespread use of pyrethroids in LLINs has led to increasing resistance of the *Anopheles* population to these compounds, with insecticide resistance (IR) being reported from several significant malaria vectors in Indonesia, including *Anopheles sundaicus, Anopheles aconitus, Anopheles subpictus,* and *Anopheles vagus* [10–12].

Insecticide resistance in mosquitoes is often mediated by two broad mechanisms: gene target-site mutations and enhanced metabolic detoxification of insecticides [13, 14]. Knockdown resistance (*kdr*) is a mechanism where target site insensitivity due to point mutations in the insect voltage-gated sodium channel (VGSC) regulatory protein, which blocks pyrethroid and Dichloro-Diphenyl–Trichloroethane (DDT) action, is associated with resistance [15]. The *Anopheles kdr* resistance allele, first detected in *Anopheles gambiae* populations from West Africa [16], is due to a single amino acid substitution (leucine to phenylalanine) at nucleotide position 1014 of the gene. An alternate substitution from leucine to serine at the same position was also detected from *Anopheles* in East African Kenya [17]. West African L1014F (*kdr-w*) and East African L1014S (*kdr-e*), respectively, have been found in other geographies since their discovery [18–20].

IRS and LLINs kill susceptible mosquitoes on contact [21, 22] while SRs are designed to repel mosquitoes away from human hosts [23, 24]. Since low doses of the SR AI may not result in mortality, selection for resistance in the mosquito should be diminished [24–27]. The evaluation of a transfluthrin-based SR product in Sumba, Indonesia, for protective efficacy against malaria [28, 29] enabled this temporal evaluation of the frequency of the *kdr* insecticide resistance allele in *Anopheles* before and after the implementation of the trial. The results of this study are the first report regarding the existence of *kdr* allele in *Anopheles* species in Sumba, Indonesia.

## Methods

### Ethic statement

This study was approved by the Ethics Committee of Research in Health, Medical Faculty of Hasanuddin University, Makassar, Indonesia No: 01641/H4.8.4.5.31/PP36-KOMETIK/2014; 0424/H4.8.4.5.31/PP36-KOMETIK/2015; 225/H4.8.4.5.31/PP36-KOMETIK/2016 AND 923/H4.8.4.5.31/PP36-KOMETIK/2017.

### Study area, study design, and survey periods

The study was conducted in West Sumba and Southwest Sumba Districts, East Nusa Tenggara. The population in the study villages mostly work in agriculture and live in traditional houses made of bamboo. In Southwest Sumba, out of 52.8% from total land area used for agriculture with 6.2% of total land area is used for paddy field, while 46.7% is used for cropland. Meanwhile in West Sumba, out of the 86% of the area used for agriculture included 10.6% for paddyfield and 75.6% for cropland. These agricultural lands are dispersed throughout the clusters area [30, 31].

The parent spatial repellent efficacy study was a cluster-randomized, double-blinded, placebo-controlled trial involving with a total 24 clusters divided into 12 cluster per treatment arms (treated or placebo) [28]. Entomologic survey was performed in 3 periods: baseline, intervention, and post-intervention. The baseline is the phase before SR product distribution (June 2015–March 2016); the intervention was during SR product deployment (April 2016–April 2018), and post-intervention was the phase after intervention where no SR was used. Adult mosquito diversity and densities were measured using the Human Landing Catch (HLCs) every 2 weeks from the start of the baseline through the end of the follow-up intervention period. The intervention was launched simultaneously in all clusters and study personnel was distributed the SR product; transfluthrin-based passive emanator produced by S.C. Johnson & Son, Inc. (SCJ). Spatial repellent products were positioned indoor by hung on two metal hooks specially attached to walls. Research staff placed, removed, and replaced SR products in households every 2 weeks.

Susceptibility assays were conducted using CDC bottle assay. Three different insecticides were evaluated using adult F0 anopheline species collected as immatures from larval collection in each cluster in baseline, intervention, and post–intervention periods. Representative samples from susceptibility assay (intervention and post–intervention) and HLCs collection (baseline and intervention) were analysed for the *kdr* allele [28].

### Mosquito larval sampling

All potential *Anopheles* breeding sites within the target cluster were sampled, and coordinates were recorded. Early stages (1st and 2nd instars) were discarded, and late-stage larvae (3rd and 4th instars) and pupae were counted, recorded, and transported to the field insectary. Larvae were fed daily with a mixture of finely ground fishmeal and yeast and reared to adults. Adult females were transferred to cages (40 cm^3^ metal frame covered with untreated mosquito nets), held for 3–5 days, and fed a sugar solution until use in bioassays.

### Adult mosquito collection

Adult female mosquitoes were collected using HLCs and collections were conducted in 12 spatially distributed geographic clusters, using paired (indoors and outdoors) volunteer collectors in four selected houses at each collection site. Host–seeking mosquitoes landing on exposed feet and legs were caught using an aspirator for 50 min each hour from 18.00 to 06.00 h. Mosquitoes were held in individual paper cups labeled for each hour, location (indoor or outdoor), and household. Mosquito specimens were morphologically identified to species using taxonomic keys [32].

### Insecticide susceptibility assay

The standard dosages or concentrations that were applied were recommended by the Centers for Disease Control (CDC) and the World Health Organization (WHO), i.e. deltamethrin 12.5 μg/ml, and permethrin 21.5 μg/ml, with a knockdown (KD) time of 30 min. Unlike the other two insecticides, previously transfluthrin was tested on wild-type *An. aconitus* laboratory strain at various concentrations (10, 12, 14, 16 μg/ml) before reaching a dose of 10 μg/ml with a KD time of 35 min [33]. A CDC bottle assay was performed by coating glass bottles with insecticide technical grade solution, exposing mosquitoes, and observed for 2 h. The assays were conducted using non–bloodfed, 3–5 day old females according to established guidelines [33]. Resistance status is determined by the percentage of mortality rate after 2 h observation. After each test period, all specimens were stored individually over silica gel for molecular analysis.

### DNA extraction

Homogenate and mosquito DNA isolation from individual Anopheline were prepared following cetyl trimethyl ammonium bromide (CTAB) 2% reagent protocol. The CTAB technique was performed according to described protocols [34, 35]. Briefly, mosquitoes were ground with pestles in 1.5 ml microtubes containing 200 μl 2% CTAB and vortexed for 15 s, and then incubated in a heating block at 65 °C for 20 min, after which 200 μl of chloroform was added to each sample and mixed by vortex for 15 s and centrifuged at 12,000 RPM for 5 min. The aqueous phase was transferred to new vials (1.5 ml), 100 μl of cold isopropanol (− 12 °C) was added, and then samples were stored at − 30 °C for 15 min. Following incubation, the samples were centrifuged at 13,000 RPM for 5 min. The supernatant was then decanted, followed by adding 100 μl of cold 70% ethanol (− 12 °C) and centrifugation at 12,000 RPM for 5 min. The ethanol was then decanted, and the DNA pellets were dried in the vial. Finally, the pellets were resuspended by adding 20 μl of water. DNA was used immediately for a polymerase chain reaction (PCR) or stored at − 20 °C for later analysis.

### Gene amplification with PCR and sequencing of *kdr* loci

The *kdr* gene was amplified using three primers in a semi-nested PCR [11]. Amplified products were cleaned using Exosap and sequenced using Sanger technology with an ABI BigdyeTM terminator per the manufacturer’s recommendation.

### Analysis

Sequences were submitted to the National Center for Biotechnology Information’s Basic Local Alignment Search Tool (https://blast.ncbi.nlm.nih.gov/Blast.cgi) to blast and confirm that the correct loci were amplified. Sequences were then aligned in locus target to identify *kdr* L1014F or L1014S mutations based on a reference sequence. Hardy–Weinberg equilibrium for observed genotyped frequencies for each *kdr* mutation was calculated using GenAlEx 6.5 [36]. Statistical analysis using the Pearson's Chi-squared test was conducted to compared *kdr* allele frequency in clusters with placebo and treated in the baseline and intervention periods.

## Results

### Larval samples

#### Habitat description and species composition

**Fig. 1:**
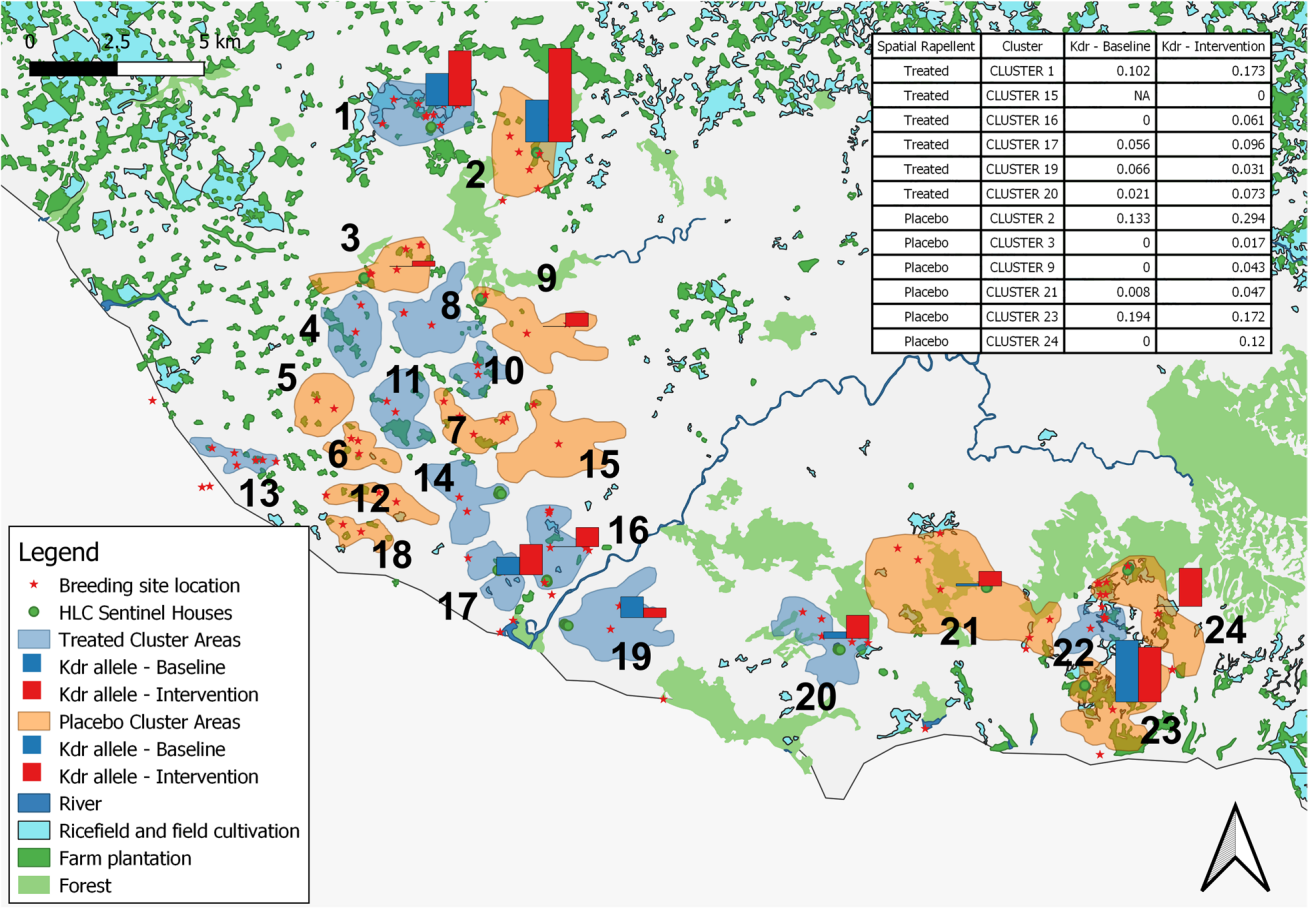
Map of the study site: Breeding site location in clusters, positive Anopheles larvae are marked by a red star

Larval collections in the study sites (Fig. 1) were conducted across all three time points. Natural larval sites were more prevalent than artificial sites. *Anopheles* larvae have been discovered in paddy fields, streams, seepage, ground pools, and estuaries (Additional file 1). Morphologically identified samples included 12 *Anopheles* species, including *Anopheles tesselatus, Anopheles aconitus, Anopheles barbirostris, Anopheles kochi, Anopheles vagus, Anopheles annularis, Anopheles maculatus Anopheles sundaicus, Anopheles flavirostris, Anopheles balabacensis, Anopheles indefinitus* and *Anopheles subpictus. Anopheles vagus* was the most abundant species in all larval sites studied and, therefore, proportionally tested in insecticide assays, whereas *An. sundaicus* was dominant in estuaries.

#### Insecticide susceptibility bioassays

In total, 674 female *Anopheles* mosquitoes reared from larvae were evaluated for insecticide susceptibility with CDC bottled assay. Due to variations in larval numbers, as well as time to pupation and emergence, the number of replicates per insecticide and the number of mosquitoes per assay varied. The number of individuals used in any single assay ranged from 10 to 25, and the minimum number of replicates was four. The results from the test using deltamethrin (baseline), transfluthrin (intervention), and permethrin (post-intervention) demonstrated that Anopheline mosquitoes were susceptible to all three pyrethroids, with a 100% mortality rate (Table 1).

**Table 1:** Summary results of the bioassays with 3 insecticides

Study period	Insecticides	n^a^	% Mortality^b^	% KDT^c^	% Control^d^	Status^e^
Baseline	Deltamethrin, 12.5 μg/ml	84	100	78.6	100	S
Intervention	Transfluthrin, 10 μg/ml	238	100	99.6	100	S
Post-intervention	Permethrin, 21.5 μg/ml	352	100	100	100	S

^a^n: number of mosquitoes examined

^b^% mortality: mortality rate (expressed in %) after 2 h of exposure to insecticide

^c^KDT: rate of mosquitoes “knocked down” (KD, expressed in %) recorded at diagnostic time. 30 min for deltamethrin and permethrin, 35 min for transfluthrin

^d^% control: survival rate of the mosquito group that did not receive insecticide (expressed in %) calculated after 2 h observation

^e^Status: resistance status as defined by WHO. Briefly, a mortality in the range 98–100% indicates susceptibility (S); a mortality between 90 and 97% indicates suspected resistance; a mortality < 90% indicates resistance (R)

#### Allele and genotype frequencies of the *kdr* 1014 mutation

Mosquitoes (intervention and post-intervention period) from the susceptibility assay (n = 890) were analyzed by sequencing the *kdr* allele. The *kdr* allele—wild type (TTA/TTA, TTA/TTG and TTG/TTG), mutant allele L1014F (TTT/TTA, TTT/TTG, TTT/TTT) and L1014S (TCA/TTA and TCA/TCA) were identified. The L1014F (*kdr-w*) allele was detected in *An. sundaicus*, *An. subpictus, An. tesselatus*, *An. vagus* and *An. annularis.* The L1014S (*kdr-e*) allele was detected in *An. sundaicus*, *An. vagus* and *An. annularis*. In addition to these alleles, a heteroduplex two amino acid substitution at 1014 position was documented in *An. sundaicus, An. vagus,* and *An. tesselatus.* The *kdr* genotype frequency increased from 0.059 to 0.203 with *An. tesselatus* as the spesies that saw an increase in the *kdr* allele (Table 2).

**Table 2:** Frequency of *kdr* allele in *Anopheles* raised from larvae

Species	INTERVENTION								POST INTERVENTION							Frequency R allele
	n	Wild-type	Mutant					Frequency R allele	n	Wild-type	Mutant					
		SS	L1014F		L1014S		Hetero duplex			SS	L1014F		L1014S		Hetero duplex	
			RS	RR	RS	RR					RS	RR	RS	RR		
*An. aconitus*	21	21	–	–	–	–	–	0.000	26	26	–	–	–	–	–	0.000
*An. sundaicus*	303	280	5	3	1	7	7	0.066	63	62	–	–	–	–	1	0.016
*An. vagus*	184	177	–	–	–	5	2	0.038	82	81	–	–	–	1	–	0.012
*An. subpictus*	21	19	–	1	–	–	1	0.095	57	57	–	–	–	–	–	0.000
*An. barbirostris*	11	11	–	–	–	–	–	0.000	17	17	–	–	–	–	–	0.000
*An. tesselatus*	8	3	2	3	–	–	–	0.500	70	1	16	–	–	50	3	0.871
*An. annularis*	–	–	–	–	–	–	–	#NA	5	1	1	–	–	3	–	0,700
*An. kochi*	–	–	–	–	–	–	–	NA	8	8	–	–	–	–	–	0.000
*An. maculatus*	14	14	–	–	–	–	–	0.00	–	–	–	–	–	–	–	NA
Total	562	525	7	7	1	12	10	0.059	328	253	17	-	-	54	4	0.203

n: the total number of samples examined. Zero sample is indicated by ‘–‘

^#^*NA* Not applicable, *SS* Susceptible, *RS* Heterozygous resistant, *RR* Homozygous resistant

### Adult samples

#### *kdr* baseline and intervention phase

Overall, 3520 adult female Anopheles mosquitoes from the six treated and six placebo clusters were analyzed from the baseline and intervention phases. The *kdr* allele (L1014F/S) were detected in 11 morphologically and molecularly identified species, namely *An. aconitus, An. maculatus, An. sundaicus, An. vagus, An. subpictus, An. barbirostris, An. tesselatus, An. annularis, An. flavirostris, An. indefinitus,* and *An. kochi.* In general, all species showed an increase in the *kdr* allele during the intervention, except *An. subpictus* from treated arm, and *An. sundaicus* from placebo arm. However, *Anopheles* species from placebo arm have a higher *kdr* frequency (Table 3).

**Table 3:** Frequency of *kdr* allele in each *Anopheles* species from adult mosquito collection

Arms	Species	Baseline								Intrevention							
		n	Wild-type	Mutant					Frequency of R allele	n	Wild-type	Mutant					Frequency of R allele
			SS	L1014F		L1014S		Hetero duplex			SS	L1014F		L1014S		Hetero duplex	
				RS	RR	RS	RR					RS	RR	RS	RR		
Treated	*An. aconitus*	81	79	2	–	–	–	–	0.012	733	678	24	19	9	2	1	0.053
	*An. annularis*	21	13	2	–	5	–	1	0.214	37	21	2	1	8	2	3	0.297
	*An. balabacensis*	–	–	–	–	–	–	–	#NA	–	–	–	–	–	–	–	NA
	*An. barbirostris*	4	4	–	–	–	–	–	0.000	19	18	–	1	–	–	–	0.053
	*An. flavirostris*	92	86	6	–	–	–	–	0.033	327	306	14	4	3	–	–	0.038
	*An. indefinitus*	–	–	–	–	–	–	–	NA	4	4	–	–	–	–	–	0.000
	*An. kochi*	2	2	–	–	–	–	–	0.000	33	30	1	2	–	–	–	0.076
	*An. maculatus*	13	13	–	–	–	–	–	0.000	61	58	3	–	–	–	–	0.025
	*An. subpictus*	2	–	–	–	–	2	–	1.000	6	3	1	–	–	1	1	0.417
	*An. sundaicus*	157	154	2	–	–	1	–	0.013	19	18	–	–	–	1	–	0.053
	*An. tesselatus*	11	7	2	2	–	–	–	0.273	53	26	12	11	1	–	3	0.387
	*An. vagus*	40	39	–	1	–	–	–	0.025	94	83	3	2	2	2	2	0.090
	All Anopheles	423	397	14	3	5	3	1	0.039	1386	1245	60	40	23	8	10	0.072
Placebo	*An. aconitus*	17	17	–	–	–	–	–	0.000	59	49	3	5	2	–	–	0.127
	*An. annularis*	85	66	2	2	14	1	–	0.129	138	84	10	4	33	6	1	0.236
	*An. balabacensis*	–	–	–	–	–	–	–	NA	18	18	–	–	–	–	–	0.000
	*An. barbirostris*	16	15	1	–	–	–	–	0.031	60	54	1	3	–	–	2	0.092
	*An. flavirostris*	76	75	–	–	–	–	1	0.013	166	157	3	4	1	–	1	0.042
	*An. indefinitus*	–	–	–	–	–	–	–	NA	8	3	–	2	–	2	1	0.625
	*An. kochi*	64	61	2	–	–	–	1	0.031	223	197	10	8	4	4	–	0.085
	*An. maculatus*	17	10	5	2	–	–	–	0.265	33	32	–	–	1	–	–	0.015
	*An. subpictus*	3	2	–	–	–	–	1	0.333	55	16	2	6	4	10	17	0.655
	*An. sundaicus*	27	24	1	–	2	–	–	0.056	12	12	–	–	–	–	–	0.000
	*An. tesselatus*	43	20	8	14	–	–	1	0.442	240	79	43	106	3	1	8	0.575
	*An. vagus*	119	112	2	3	2	–	–	0.042	232	195	6	4	1	9	17	0.144
	All *Anopheles*	467	402	21	21	18	1	4	0.097	1244	896	78	142	49	32	47	0.191

n: the total number of samples examined. Zero sample is indicated by ‘–‘

^#^*NA* Not applicable, *SS* Susceptible, *RS* heterozygous resistant, *RR* homozygous resistant

Pearson's Chi-squared test with Yates' continuity correction revealed there was a significant difference meaning between mutant and wildtype numbers in the treated cluster (p = 0.0126), placebo cluster (p = 2.105e-10) and treated vs placebo clusters (p-value < 2.2e-16) during baseline and intervention. The intervention period samples had a higher frequency of all mutant alleles than baseline samples. The frequency of mutant alleles increased 1.1 times from 0.070 to 0.146 (Table 4).

**Table 4:** Frequency of *kdr* allele in each cluster from adult mosquito collections

Baseline										Intervention							
Arms	Cluster	n	Wild-type	Mutant					Frequency of R allele	n	Wild-type	Mutant					Frequency of R allele
			SS	L1014F		L1014S		Hetero duplex			SS	L1014F		L1014S		Hetero duplex	
				RS	RR	RS	RR					RS	RR	RS	RR		
Treated	01	64	55	3	3	2	–	1	0.102	130	101	5	8	8	2	6	0.173
	15	–	–	–	–	–	–	–	#NA	4	4	–	–	–	–	–	0.000
	16	107	107	–	–	–	–	–	0.000	717	651	37	18	8	1	2	0.061
	17	18	16	–	–	2	–	–	0.056	114	100	4	4	2	2	2	0.096
	19	91	82	5	–	1	3	–	0.066	196	187	6	2	–	1	–	0.031
	20	143	137	6	–	–	–	–	0.021	225	202	8	8	5	2	–	0.073
Placebo	02	312	253	19	21	16	1	2	0.133	854	551	65	131	39	28	40	0.294
	03	20	20	–	–	–	–	–	0.000	87	85	–	1	1	–	–	0.017
	09	14	14	–	–	–	–	–	0.000	46	44	–	–	–	–	2	0.043
	21	63	62	1	–	–	–	–	0.008	74	69	3	2	–	–	–	0.047
	23	18	13	1	–	2	–	2	0.194	87	65	7	2	7	3	3	0.172
	24	40	40	–	–	–	–	–	0.000	96	82	3	6	2	1	2	0.120
Total		890	799	35	24	23	4	5	0.070	2630	2141	138	182	72	40	57	0.146

n: the total number of samples examined. Zero sample is indicated by ‘–‘

^#^*NA* Not applicable, *SS* Susceptible, *RS* Heterozygous resistant, *RR* Homozyous resistant

The mean of *kdr* allele frequency in the placebo cluster, baseline (0.056 ± 0.086), and intervention (0.116 ± 0.104) were higher than SR treated clusters, placebo (0.049 ± 0.04) and intervention (0.072 ± 0.059).

## Discussion

A growing body of evidence has demonstrated the potential use of SRs to control malaria and other mosquito-borne diseases [28, 37]. With resistance to active ingredients (AIs) used on intervention products being a significant factor that compromises intervention effectiveness, surveillance for IR is a vital component of any evaluation of an intervention—especially novel paradigms such as SRs. Surveillance for phenotypic IR and *kdr* alleles was conducted before, during, and after the parent study that evaluated the impact of a pyrethroid (transfluthrin)-based SR product on malaria incidence in which clusters of households were treated with either a transfluthrin-based SR or a placebo.

Phenotypic IR surveillance for IR demonstrated that all *Anopheles* species in the study clusters remained fully susceptible to multiple pyrethroids following the 2 year SR intervention in Sumba, Indonesia. Molecular analysis revealed that the *kdr* resistance allele was already present in the population at baseline. However, various alleles increased in frequency over the study sites throughout the primary study.

Chemical pesticides, including pyrethroids, are the primary focus for agricultural pest management for fruit and vegetable crops in Sumba Islands including corn as the main crop followed by paddy field. In addition, as an area endemic to malaria, public health insecticide use is often intense. Though the use of insecticides in agriculture is generally regarded as an important driver of insecticide resistance in malaria vectors [38], pyrethroid-based interventions (ITNs and IRS) for controlling *Anopheles* have been associated with an increase in the frequency of *kdr* resistance alleles [39, 40]. Here, agricultural use of pyrethroids, along with pyrethroid-based LLINs, likely enabled the selection of baseline *kdr* resistance alleles seen at baseline. The increased frequency of *kdr* in placebo clusters is likely due to local selection by agricultural insecticides and chance inclusion in the placebo arm. Mass distribution of LLINs in Sumba occurred in October–December 2014 (Olyset Net, permethrin 2.0%) with reported > 95% coverage of households providing 1–4 nets each. Another mass distribution occurred in February–March 2018 (PermaNet^®^ 3.0, Vestergaard Frandsen SA, Denmark, deltamethrin 180 mg/m^2^ + piperonyl butoxide synergist) [28]. The increased selection for *kdr* seen in untreated arms due to factors external to SR implementation is supported by the temporal more significant increase in these alleles from the baseline to the intervention periods in these clusters without SRs being implemented.

These data support the SR paradigm—where sublethal doses of repellant products will diminish the selection of IR phenotypes and genotypes. However, this does not counter the possibility of the emergence of SR-based resistance [23]. The selection of reduced spatial repellency, IR alleles, and reduced spatial repellant sensitivity suggests that long-term use of SRs might temporally impact vector populations. The *kdr* trait enables resistance to pyrethroid and DDT [41, 42] by reducing neuronal sensitivity [43]. This decreases the irritant and the repellent effects and either cancels or reduces the knock-down effect [44]. Here, increased *kdr* may result in less repelled mosquitoes and therefore, obtain higher doses of the volatile insecticide*,* increasing the death rate [45]. Additional secondary impacts of exposure to sublethal amounts of a spatial repellant (reduced feeding, increased mortality [46], combined with the increased lethality based on increased exposure would also support the relatively lower increase in *kdr* alleles seen in the intervention clusters in a panmictic population of mosquitoes. Unfortunately, adult collections in the post-intervention period, were not conducted, and comparisons of *kdr* allele frequencies after SR trial conclusion were impossible.

The lack of phenotypic resistance over the three time points suggests that the SR intervention did not select for pyrethroid resistance in 2 years of SR implementation. However, the three insecticides evaluated for resistance may have different insecticidal properties and were not assessed at the same time. Ideally, all three insecticides (deltamethrin at baseline), transfluthrin during the intervention, and permethrin post-intervention) would have been evaluated at all three-time points to enable a comparable evaluation of temporal insecticide susceptibility.

Of the 12 *Anopheles* species that were found to carry the *kdr* allele, 11 species are reported as human night-biting mosquitoes in Sumba [47] and confirmed as malaria vectors [10, 28] Homozygous, heterozygous, and heteroduplex forms of *kdr* alleles were found in these species and likely reflect different molecular evolution dynamics combined with different insecticide exposure levels in each *Anopheles* species. The dynamics of resistance acquisition and spread may be linked to many factors, including the history of insecticide use, the level of pre-existing susceptibility to the compound, the frequency and heritability of genes related to resistance, and the co-selection of distinct resistance mechanisms in the same population [13].

Heteroduplexes are formed between double-stranded DNA from two gene alleles [48]. The presence of a heteroduplex mutation that contains a sequence mismatch (mutant, wild-type, and wild-type, mutant) can be separated by denaturing high-performance liquid chromatography (HPLC), enzymatic (RNase cleavage assay), electrophoretic methods and chemical cleavage assays [49]. In these studies, mosquito samples with heteroduplex form were not subjected to the further examination but were still included in the resistant heteroduplex criteria carrying the mutant allele.

This study recognizes several limitations, first, during the study period, a susceptibility test of the same insecticide would be ideal. Furthermore, this resistance status may be restricted by location, species or influenced by the number of samples tested in each location, requiring testing in each cluster. Second, adult mosquito collection in post-intervention should be performed to confirm the frequency of the *kdr* mutant allele in the absence of SR. Third, detecting metabolic detoxification enzymes will contribute to comprehensively understanding the causes of the high level of mutant *allele* in *Anopheles* populations in Sumba. Overall, the concurrent use of pyrethroids in public health and agricultural sectors continues to drive mosquito resistance and alerts to the need to use alternative tools that do not drive resistance. It is also essential to mitigate the emergence of resistance by applying different classes of insecticides for public health and agricultural purposes. In this context, sustainable insecticide resistance monitoring using phenotypic bioassays and molecular tools is necessary to inform policy and to establish an integrated vector and pest control programme. Since the Indonesian malaria control and elimination programme targets elimination by 2030, SRs may be considered for strategy inclusion as the data demonstrate an epidemiological impact [28].

## Conclusion

Implementing a transfluthrin-based SR product over 2 years did not select for resistance to pyrethroids tested. Although the *kdr* resistance allele was present at baseline and increased throughout the study, the data presented cannot explain an association with the SR. The possible external driver of *kdr* allele increases, the possible lower increase of *kdr* in intervention clusters, and the actual reduction in malaria in intervention clusters all support SRs as valid intervention in these settings.

### Supplementary Information

**Additional file 1: **Number of Anopheles larvae in breeding sites.

## Data Availability

All relevant data are within the manuscript.
