# Supplementary material for: Impact of a spatial repellent intervention on Anopheles kdr insecticide resistance allele in Sumba, Indonesia
Source: Malar J. 2024 Jan 22;23:31. doi: 10.1186/s12936-024-04841-1 (PMC10802001; doi:10.1186/s12936-024-04841-1)
Supplement: Supplementary file 1 — Additional file 1: Number of Anopheles larvae in breeding sites. [file 12936_2024_4841_MOESM1_ESM.docx]

| Spesies | Paddy field | | | | | | | | Stream | | | | | | | | |
| --- | --- | --- | --- | --- | --- | --- | --- | --- | --- | --- | --- | --- | --- | --- | --- | --- | --- |
|  | Cl. 01 | Cl. 02 | Cl. 03 | Cl. 16 | Cl. 21 | Cl. 22 | Cl. 24 | Total | Cl. 1 | Cl. 02 | Cl. 03 | Cl. 16 | Cl. 20 | Cl. 21 | Cl. 22 | Cl. 24 | Total |
| *An. aconitus* | 5 | 0 | 0 | 0 | 0 | 17 | 1 | 23 | 189 | 53 | 273 | 433 | 0 | 416 | 107 | 14 | 1485 |
| *An. sundaicus* | 0 | 0 | 0 | 14 | 0 | 0 | 0 | 14 | 0 | 0 | 0 | 0 | 0 | 0 | 0 | 0 | 0 |
| *An. vagus* | 319 | 875 | 70 | 1 | 8 | 538 | 238 | 2049 | 270 | 366 | 232 | 427 | 0 | 499 | 374 | 865 | 3033 |
| *An. subpictus* | 0 | 1 | 0 | 8 | 0 | 0 | 0 | 9 | 0 | 0 | 160 | 1 | 0 | 257 | 2 | 93 | 513 |
| *An. barbirostris* | 66 | 45 | 0 | 0 | 0 | 39 | 16 | 166 | 247 | 391 | 184 | 2 | 0 | 118 | 171 | 487 | 1600 |
| *An. tesselatus* | 225 | 92 | 1 | 0 | 0 | 1 | 1 | 320 | 0 | 22 | 0 | 0 | 0 | 1 | 0 | 0 | 23 |
| *An. annularis* | 7 | 39 | 0 | 0 | 2 | 85 | 2 | 135 | 13 | 18 | 1 | 0 | 0 | 4 | 29 | 91 | 156 |
| *An. kochi* | 100 | 207 | 65 | 1026 | 0 | 5 | 64 | 1467 | 0 | 144 | 1 | 0 | 0 | 2 | 18 | 1 | 166 |
| *An. maculatus* | 0 | 4 | 0 | 0 | 0 | 0 | 0 | 4 | 14 | 0 | 3 | 0 | 0 | 62 | 0 | 9 | 88 |
| *An.flavirostris* | 0 | 3 | 0 | 0 | 0 | 2 | 1 | 6 | 147 | 13 | 560 | 272 | 1 | 1238 | 203 | 220 | 2654 |
| *An.balabacensis* | 0 | 0 | 0 | 0 | 0 | 0 | 0 | 0 | 0 | 0 | 7 | 0 | 0 | 0 | 0 | 0 | 7 |
| *An. indefinitus* | 0 | 0 | 0 | 1 | 0 | 0 | 0 | 1 | 0 | 0 | 0 | 0 | 0 | 0 | 0 | 0 | 0 |
| Total | 722 | 1266 | 136 | 1050 | 10 | 687 | 323 | 4194 | 880 | 1007 | 1421 | 1135 | 1 | 2597 | 904 | 1780 | 9725 |

| Spesies | Ground pool | | | | | Seepage | | | | | | | | | | Estuary | | | |
| --- | --- | --- | --- | --- | --- | --- | --- | --- | --- | --- | --- | --- | --- | --- | --- | --- | --- | --- | --- |
|  | Cl. 4 | Cl. 13 | Cl. 16 | Cl. 24 | Total | Cl. 2 | Cl. 3 | Cl. 5 | Cl. 7 | Cl. 9 | Cl. 16 | Cl. 19 | Cl. 20 | Cl. 21 | Total | Cl. 13 | Cl. 19 | Cl. 23 | Total |
| *An. aconitus* | 0 | 6 | 0 | 0 | 6 | 0 | 10 | 0 | 1 | 3 | 448 | 191 | 1656 | 0 | 2309 | 0 | 5 | 0 | 5 |
| *An. sundaicus* | 0 | 0 | 0 | 0 | 0 | 0 | 0 | 0 | 1 | 0 | 0 | 0 | 0 | 0 | 1 | 1 | 722 | 811 | 1534 |
| *An. vagus* | 1 | 799 | 13 | 33 | 846 | 19 | 637 | 1 | 2037 | 814 | 83 | 0 | 157 | 0 | 3748 | 46 | 369 | 1018 | 1539 |
| *An. subpictus* | 0 | 8 | 0 | 0 | 8 | 146 | 0 | 0 | 149 | 190 | 19 | 0 | 212 | 0 | 716 | 2 | 108 | 0 | 110 |
| *An. barbirostris* | 1 | 14 | 0 | 0 | 15 | 121 | 26 | 0 | 1 | 0 | 2 | 0 | 3 | 0 | 153 | 7 | 169 | 68 | 244 |
| *An. tesselatus* | 0 | 0 | 0 | 0 | 0 | 0 | 0 | 0 | 0 | 1 | 0 | 0 | 6 | 0 | 7 | 0 | 0 | 0 | 354 |
| *An. annularis* | 0 | 0 | 0 | 0 | 0 | 2 | 0 | 0 | 0 | 0 | 0 | 1 | 1 | 0 | 4 | 0 | 31 | 7 | 38 |
| *An. kochi* | 0 | 1 | 30 | 4 | 35 | 4 | 0 | 0 | 3 | 1 | 0 | 0 | 1 | 0 | 9 | 0 | 1 | 0 | 1 |
| *An. maculatus* | 0 | 0 | 0 | 0 | 0 | 0 | 0 | 0 | 0 | 0 | 99 | 8 | 0 | 0 | 107 | 0 | 0 | 5 | 39 |
| *An.flavirostris* | 0 | 4 | 0 | 0 | 4 | 0 | 7 | 0 | 5 | 1 | 88 | 7 | 178 | 7 | 293 | 3 | 15 | 181 | 199 |
| *An.balabacensis* | 0 | 0 | 0 | 0 | 0 | 0 | 0 | 0 | 0 | 0 | 0 | 0 | 1 | 0 | 1 | 0 | 0 | 0 | 0 |
| *An. indefinitus* | 0 | 0 | 0 | 0 | 0 | 0 | 0 | 0 | 10 | 0 | 0 | 0 | 0 | 0 | 10 | 1 | 1 | 0 | 199 |
| Total | 2 | 832 | 43 | 37 | 914 | 292 | 680 | 1 | 2207 | 1010 | 739 | 207 | 2215 | 7 | 7358 | 60 | 1421 | 2090 | 3571 |

Cl: Cluster
